# Potential diagnostic biomarkers for immunogenic cell death in elderly female patients with ischemic stroke: identification and analysis

**DOI:** 10.1038/s41598-024-65390-w

**Published:** 2024-06-24

**Authors:** Lihua Qin, Sheng Li, Xi Cao, Tengjia Huang, Yixin Liu, Ouying Chen

**Affiliations:** 1 School of Nursing, Hunan University of Chinese Medicine, Changsha, 410208 Hunan People’s Republic of China; 2 Key Laboratory of Hunan Province for Prevention and Treatment of Integrated Traditional Chinese and Western Medicine On Cardiocerebral Diseases, Hunan University of Chinese Medicine, Changsha, 410208 Hunan People’s Republic of China

**Keywords:** Ischemic stroke, Immunogenic cell death, Diagnostic biomarkers, Older female patients, Differentially expressed genes, Computational biology and bioinformatics, Biomarkers

## Abstract

Ischemic stroke (IS) is of increasing concern given the aging population and prevalence of unhealthy lifestyles, with older females exhibiting higher susceptibility. This study aimed to identify practical diagnostic markers, develop a diagnostic model for immunogenic cell death (ICD)-associated IS, and investigate alterations in the immune environment caused by hub genes. Differentially expressed genes associated with ICD in IS were identified based on weighted gene co-expression network analysis and the identification of significant modules. Subsequently, machine learning algorithms were employed to screened hub genes, which were further assessed using Gene Ontology, Kyoto Encyclopedia of Genes and Genomes, and Gene Set Enrichment Analysis. A nomogram mode lwas then constructed for IS diagnosis, and its diagnostic value was assessed using a receiver operating characteristic curve. Finally, alterations in immune cell infiltration were assessed within patients with IS, and the pan-cancer expression patterns of hub genes were evaluated. Three hub genes associated with ICD (*PDK4, CCL20*, and *FBL*) were identified. The corresponding nomogram model for IS diagnosis could effectively identify older female patients with IS (area under the curve (AUC) = 0.9555). Overall, the three hub genes exhibit good diagnostic value (AUC > 0.8). *CCL20* and *FBL* are significantly associated with the extent of immune cells infiltration. Moreover, a strong link exists between hub gene expression and pan-cancer prognosis. Cumulatively, these results indicate that ICD-related hub genes critically influence IS progression in older females, presenting novel diagnostic and therapeutic targets for personalized treatment.

## Introduction

Annually, stroke claims the lives of approximately 6 million people globally and permanently disables an additional 5 million, making it a top contributor to death and disability worldwide^1^. Although it is conventionally believed that males are more susceptible to stroke, evidence suggests that afflicted females often face greater challenges during the recovery phase^2^. Specifically, females tend to exhibit poorer recovery outcomes and significantly higher disability rates compared to their male counterparts. This disparity can be attributed to myriad factors, including distinct physiological traits, hormonal influences, and societal roles inherent to females^3,4^. Meanwhile, current therapeutic and rehabilitative strategies fail to account for sex- and age-related differences. Hence, the adoption of a generalized treatment and rehabilitation approaches may exacerbate the difficulties encountered by elderly female patients, ultimately resulting in a less favorable prognosis and increased risk of recurrence^5^. To rectify this situation, researchers have undertaken in-depth investigations into the impact of sex and age on stroke prognosis to inform targeted therapeutic recommendations^6^. The ultimate goals of this research field are to devise more precise and individualized treatment plans to enhance the prognosis of elderly female patients, effectively mitigate their risk of recurrence, and improve their overall quality of life.

Immunogenic cell death (ICD) represents a distinct mode of regulatory cell demise elicited by various stressors, leading to inflammation and adaptive immune system^7^. ICD is characterized by the release or upregulation of damage-associated molecular patterns, precursor antigens, proinflammatory cytokines, and inflammatory mediators^8^. Hence, it is relatively common in certain autoimmune diseases, such as rheumatoid arthritis, in which the immune system mistakenly recognizes healthy body tissue as foreign, leading to persistent inflammation and tissue damage. Inflammatory responses play a significant role in IS pathophysiology, affecting its onset and disease progression^9^.

Ischemic brain injury can lead to secondary brain damage and neuronal death by initiating inflammatory mediator release and triggering an immune response^10^. Indeed, Immune reactions and inflammation are closely linked to stroke pathogenesis and outcomes. Moreover, recent investigation has uncovered a close association between ICD and IS incidence and progression^11^. It is, therefore, necessary to investigate ICD in older female populations with IS by screening critical targets related to ICD and identifying the underlying mechanisms behind ICD-associated IS. Ultimately, these findings may be used to improve prognosis and quality of life outcomes in IS.

This study aimed to explore, via bioinformatics, the role of ICD in IS progression among older females to improved targeted therapies. First, we identified hub target genes linked to ICD-associated IS are identified. Using these target genes, mechanisms, and immune environment alterations induced by ICD during IS development are investigated. Subsequently, a diagnostic nomogram incorporating the identified target genes is developed to assess the diagnostic utility of ICD-related biomarkers in patients with IS. Ultimately, the findings of this study enhance our understanding of IS pathogenesis, facilitating the identification of novel treatment targets and exploration of effective preventive and therapeutic strategies for IS.

## Materials and methods

### Data collection

The data utilized are freely available from the Gene Expression Omnibus (GEO) database (http://www.ncbi.nlm.nih.gov.hnucm.opac.vip/). The GSE37587 dataset related to IS comprised 28 peripheral blood samples from females aged > 60 years with IS. Additionally, the GSE22255 dataset related to IS included peripheral blood samples from 5 females aged > 60 years with IS and 5 age-matched healthy female controls.

Meanwhile, the GSE22255 dataset related to IS included peripheral blood samples from 5 females aged > 60 years with IS and 5 age-matched healthy female controls. Furthermore, the GSE16561 dataset contained 16 peripheral blood samples from females aged > 60 years with IS and 6 age-matched healthy female controls. The GSE58294 dataset served as an external validation set in the current study and included 11 peripheral blood samples from women aged > 60 years with IS and 11 age-matched healthy control samples. We utilized the ComBat method within the “sva” package in R software to correct batch effects caused by non-biological factors^12^. The effectiveness of this correction was assessed using principal component analysis. Genes associated with ICD were obtained from Garg et al.^13^ (Supplementary Material Table S1).

### Differentially expressed genes associated with IS

Utilizing the R package “limma” (v3.50.0)^14^, we identified differentially expressed genes (DEGs) between control (n = 11) and experimental (n = 49) groups, applying strict criteria of |log2Fold Change|> 1 and *p* < 0.05. The resulting DEGs were further analyzed to understand their biological relevance. We employed the “pheatmap” R package, utilizing Euclidean distance and hierarchical clustering, to generate a heatmap for visualizing and analyzing expression patterns in our data.

**Weighted gene co-expression network analysis and identification of significant modules**

The R package “WGCNA (version 1.70–3)” to employed to construct co-expression networks^15^. Similarity in genetic expression profiles was assessed using Pearson correlation coefficients, which were then weighted by a power function to construct a scale-free network, revealing complex gene interactions. The PickSoftThreshold method from the R package "WGCNA" was utilized to increase co- expression similarity to a power *β* = 5 to establish a weighted adjacency matrix.

Gene modules, which represent clusters of densely connected coexpressed genes, were identified through weighted gene co-expression network analysis (WGCNA) hierarchical clustering. Distinct modules were then identified using the dynamic tree-cutting method. During the module selection process, the adjacency matrix was converted into a topological overlap matrix, and clustering analysis identified the modules. The association between the module eigengenes (MEs) and ICD was calculated using Pearson correlation analysis, identifying modules significantly associated with ICD. The structures of the identified co-expression modules were visualized using a gene network topology overlap heatmap. Hierarchical cluster trees of the feature vectors and their corresponding feature vector heat maps were used to depict the relationships between the modules. Finally, DEGs associated with ICD were obtained from the intersection between the IS DEG and ICD-associated gene modules.

#### Hub gene identification

Least absolute shrinkage and selection operator (LASSO) regression was conducted to calculate and select linear models while retaining the most relevant variables, utilizing the “glmnet” package in R. Subsequently, binary distribution variables were employed for LASSO classification, and the model with the smallest error was established comprising ten cross-validation variables with good performance.

Next, the “RandomForest” function was utilized to conduct random forest analysis. The smallest error was chosen as the entry node value, and values that tended to stabilize were selected as the tree. The top 20 DEGs linked to ICD were identified using average mean decreases in accuracy and Gini as evaluation criteria. Finally, by integrating LASSO regression and random forest results, the most significant genes were identified.

#### Gene set enrichment analysis

Gene set enrichment analysis (GSEA) was conducted to determine whether predefined gene sets exhibited consistent significant differences between biological states^16^. Additionally, single-sample GSEA (ssGSEA) was used to understand the potential mechanisms by which the hub genes affect IS. Log twofold change values for other genes were calculated separately for the high and low hub gene groups. Using the “clusterProfiler” package (version 4.2.2), genes were sorted based on their log twofold change values, and GSEA was performed with 1,000 permutations. The reference gene sets selected for this study were obtained from the Molecular Signatures Database, specifically c2.cp.kegg.v7.5.1.symbols^16–18^.

#### Gene ontology and kyoto encyclopedia of genes and genomes analyses

Gene Ontology (GO) enrichment analysis^19^ was employed to assess the enrichment of GO terms within the gene sets. Kyoto Encyclopedia of Genes and Genomes (KEGG) analysis was utilized to identify enriched significant metabolic pathways from corresponding gene lists^20^. Both GO and KEGG enrichment analyses were performed using the “clusterProfiler” package (*p* < 0.05)^21^.

### GeneMANIA analysis

Relationships between functionally similar genes and hub genes were predicted using GeneMANIA online software (http://genemania.org)^22^. Utilizing GeneMANIA, we constructed a protein–protein interaction (PPI) network for the hub genes, revealing intricate interactions among them. Subsequently, the R package “clusterProfiler” was employed for GO and KEGG analyses of the hub genes and their interacting genes within the PPI network.

#### Receiver operating characteristic curve

Receiver operating characteristic (ROC) curves serve as indicators of the sensitivity and specificity of continuous variables, with the area under the curve (AUC) being the most commonly used metric of these curves. The AUC is derived from the operational characteristic plot of sensitivity and specificity for the subjects. In the current study, the “pROC” package was utilized to generate ROC curves and the AUC was calculated; this was then used to screen characteristic genes and assess their diagnostic value^23^. Overall, a higher AUC value, approaching 1, indicated a better diagnostic efficacy.

### Development and evaluation of a diagnostic nomogram for the identification of IS

A diagnostic nomogram model for IS was developed employing the R package “rms” to facilitate clinical decision-making and risk assessment. The risk score for this model was calculated based on the expression levels of hub genes, with the total risk score as the sum of the risk scores for each individual gene. The diagnostic value of the nomogram model for IS was evaluated using calibration and ROC curves. To further validate the analysis results, we conducted external validation using GSE58294 to assess the predictive performance of the diagnostic model for IS in an external independent dataset.

### Immune infiltration analysis

ssGSEA was used to calculate the enrichment scores for each sample and gene set^24^. Each ssGSEA enrichment score reflected the extent of coordinated upregulation or downregulation of genes within a specific gene set. Utilizing the Tumor-Immune System Interaction Database(http://cis.hku.hk/TISIDB/index.php)^25^, immune cell marker gene data were obtained, and relative enrichment scores were subsequently calculated for various immune cell types, providing insights into the immune cell landscape. R’s “ggplot2” (v3.3.6) was employed to plot immune cell infiltration in patients with IS versus controls, revealing immune response differences^26^.

### Pan-cancer analysis

Pan-cancer analysis was conducted using hub genes to examine their pan-cancer expression, impact on survival, and level of immune infiltration. Genomic expression profiles and clinical data for 33 cancer types were retrieved from The Cancer Genome Atlas database using the R package “TCGAbiolinks (version 2.25.0)”^26^. Differences in expression between tumor samples and normal samples were assessed using the Wilcoxon test for hub genes. For survival analysis, the impact of hub gene expression on tumor prognosis was evaluated using a univariate Cox regression model. Finally, ssGSEA was used to calculate immune infiltration scores and assess their correlation with hub gene expression, revealing potential immune-genetic interactions.

### Statistical analysis

Statistical analysis was conducted using R (version 4.1.2). Spearman’s rank correlation coefficient analysis was used to identify potential relationships. The non-parametric Wilcoxon rank-sum test was used to compare two independent samples, while the Kruskal–Wallis non-parametric test was utilized for the analysis of three or more independent sample groups. All statistical analyses used a two-tailed *p*-value < 0.05 as the criterion for significance, ensuring the rigor and reliability of the results.

## Results

The study flowchart is presented in Supplementary Fig. s1.

### Screening ICD-associated DEGs

**Figure 1:**
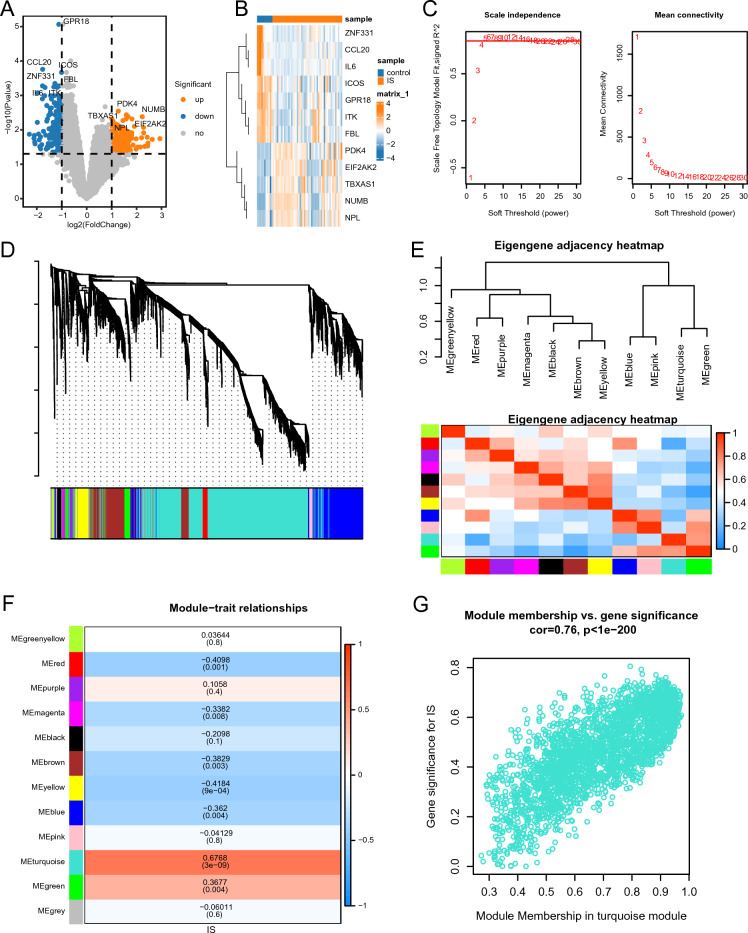
Selection of DEGs related to ICD. (**A**) Volcano plot of DEGs distribution between the IS and control groups. Red, blue, and gray dots represent upregulation, downregulation, and no significant expression, respectively. (**B**) Heatmap of top five upregulated and seven downregulated gene expression. (**C**) A soft threshold of β = 5 was selected to construct a scale-free gene expression network; the scale-free topology fitting index (R^2^) was used to assess the network’s fitness. (**D**) Analysis of the gene expression network in IS revealed distinct modules within the co-expression data. (**E**) Relationships between modules explored to gain insights into their functional interactions and regulatory mechanisms. Top: Hierarchical clustering of module-defining genes, summarizing the modules identified in the clustering analysis. The dendrogram branches (meta-modules) positively correlated feature genes together. Bottom: Correlation heatmap of feature gene networks. Each row and column corresponds to a feature gene of a module (colored for identification). Red: high degree of adjacency, blue: low degree of adjacency; red rectangles along the diagonal are indicative of meta-modules. (**F**) Relationship between consistent module-defining genes and ICD. Each row corresponds to a consistent module; each column corresponds to a trait. The numbers in represent the correlation between the feature genes of the respective module and the trait; P-values are shown below the correlation in parentheses. (**G**) Correlation between the module membership (MM) of all genes in the turquoise module and the significance of genes related to ICD. Cor: absolute correlation coefficient between gene significance and MM. DEG: differentially expressed gene; IS: ischemic stroke.

By comparing IS samples with control group samples, 329 DEGs were identified including 169 upregulated and 160 downregulated genes (Supplementary Material Table S2), which were visualized using a volcano plot (Fig. 1A). The observed.

differences between the two groups were found to be significant. The top five upregulated genes (*TBXAS1, PDK4, EIF2AK2, NUMB,* and *NPL*) and the top seven downregulated genes (*GPR18, CCL20, ICOS, ZNF331, IL6, ITK,* and *FBL*) were visualized using a heatmap (Fig. 1B).

Using WGCNA, gene sets related to ICD were identified. With a weight value of 5, the average connectivity was approximately 0, while the average scale-free index exceeded 0.85, indicating a highly organized gene co-expression network related to IC (Fig. 1C). Moreover, 11 co-expression modules were identified, all of which, excluding the gray module, contained irrelevant genes from further analysis (Fig. 1D).

Correlation analysis of the MEs provided insights regarding the relationships among modules and their potential roles in ICD. Dendrograms and heat maps were constructed to illustrate the network of characteristic genes (Fig. 1E). Eleven MEs were identified as being associated with ICD and prioritized significant associations. The heatmap analysis (Fig. 1F) showed that the turquoise module, clustering 2654 genes (Supplementary Material S3), exhibited a strong positive correlation with ICD (r = 0.6738, *p* < 0.05). Given its potential to provide a more precise indication of ICD, the turquoise module was primarily focused on in subsequent analyses.

Figure 1G illustrates the gene significance of the ICD characteristics within the turquoise module in relation to the module membership. A significant positive correlation (cor = 0.76, *p* < 0.05) was observed between module membership and gene significance of ICD characteristics in the turquoise module. By intersecting the IS-associated DEGs with the genes associated with ICD, 215 IS-associated DEGs related to ICD were identified (Supplementary Material Table S4).

### Using machine learning algorithms to identify hub genes

**Figure 2:**
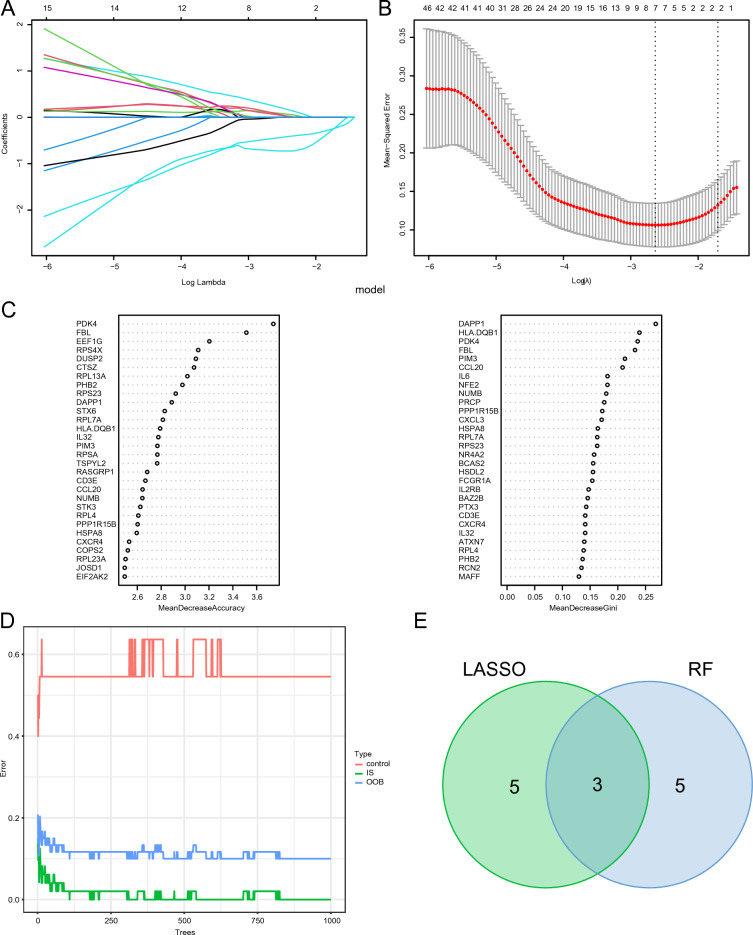
Utilizing machine learning methodologies to pinpoint potential diagnostic biomarkers that could signify IS progression. (**A**) Trajectory of independent variables in LASSO regression; x-axis: the logarithm of the tuning parameter lambda, y-axis: corresponding coefficients. (**B**) Confidence intervals at different lambda values in LASSO regression. (**C**) Top 30 DEGs related to ICD in IS, ranked by importance scores using two methods in the random forest algorithm. (**D**) Random forest error rate compared to the number of classifier trees. (**E**) Venn diagrams of hub genes.

Hub genes related to ICD were filtered using LASSO regression and random forest analysis. Notably, eight essential DEGs related to ICD were identified through LASSO regression analysis (Fig. 2A, B). Additionally, based on the feature weights of mean decrease in accuracy and mean decrease in Gini, the random forest algorithm identified the top 30 genes as key ICD-related DEGs (Fig. 2C, D). Three of the most critical IS-associated DEGs related to ICD were selected as hub genes for subsequent analysis; this was accomplished by intersecting the ICD-related DEGs identified using these two methods (Fig. 2E, Supplementary Material Table S5).

### Hub gene expression patterns and ssGSEA enrichment analysis

**Figure 3:**
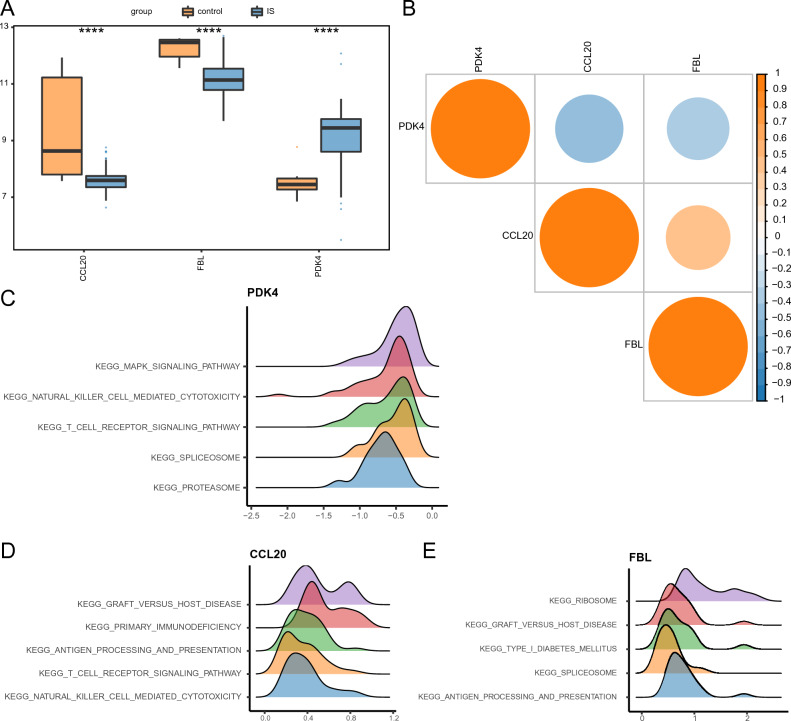
Expression patterns of hub genes and ssGSEA enrichment. (**A**) Box plot of hub gene expression patterns. (**B**) Correlation heatmap of hub genes. (**C**–**E**) ssGSEA enrichment of (**C**) *PDK4*, (**D**) *CCL20*, and (**E**) *FBL*.

The expression patterns of hub genes were analyzed using box plots and correlation heatmaps to understand their variation and relationships. Notably, the expression levels of *CCL20* and *FBL* were significantly lower in the IS group than those in the control group, while *PDK4* was significantly higher (Fig. 3A). Furthermore, *CCL20* and *FBL* displayed a strong positive correlation; in contrast, *PDK4* exhibited negative correlations with both *CCL20* and *FBL* expression (Fig. 3B).

The ssGSEA analysis results showed that genes with a similar expression pattern to *PDK4* were primarily enriched in MAPK signaling, natural killer (NK) cell–mediated cytotoxicity, T cell receptor (TCR) signaling, spliceosome, and proteasome processes (Fig. 3C). Moreover, genes with similar expression patterns to *CCL20* were primarily enriched in processes associated with graft-versus-host disease, primary immunodeficiency, antigen processing and presentation, TCR signaling, and NK cell mediated cytotoxicity (Fig. 3D). Finally, genes with expression patterns similar to *FBL* were primarily enriched in processes associated with ribosomes, graft-versus-host disease, type 1 diabetes mellitus, spliceosomes, and antigen processing and presentation (Fig. 3E).

### Analysis of hub gene interactions

**Figure 4:**
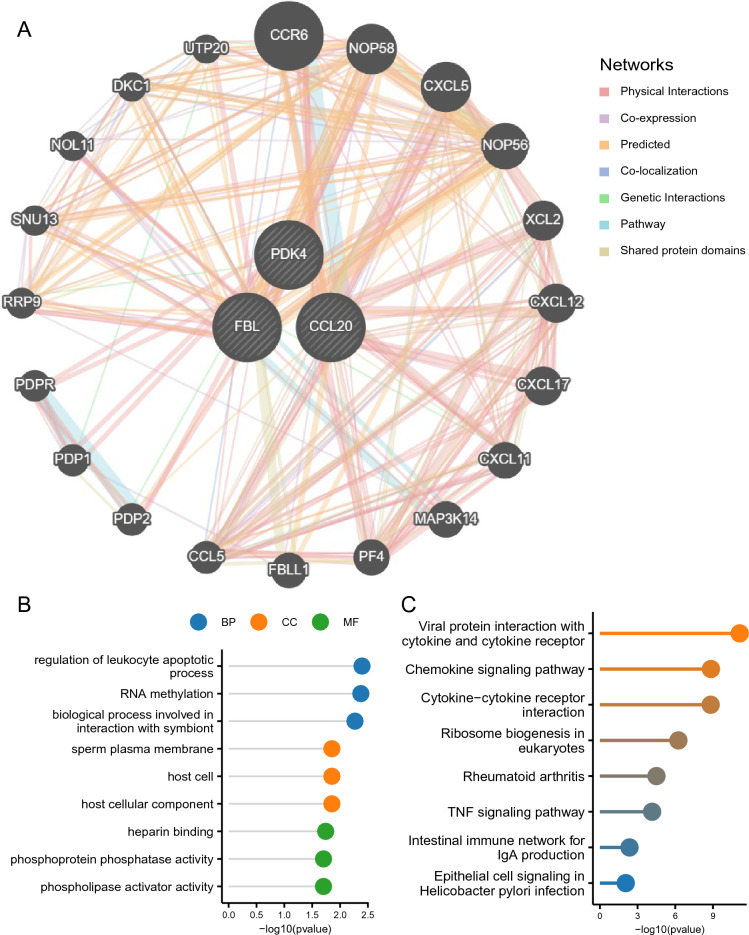
Analysis of hub gene interactions. (**A**) Gene co-expression network. (**B**) GO enrichment analysis of the coexpressed genes. (**C**) KEGG enrichment analysis of the co-expressed genes.

Using GeneMANIA, PPI networks for hub genes were constructed, revealing interactions among all three (Fig. 4A). To investigate the functions of characteristic genes, GO and KEGG analyses were performed on a set of 23 genes (3 hub genes, 20 genes associated with hub genes, and 400 connections; Supplementary Material Table S6), revealing significant enrichments in biological processes such as leukocyte apoptosis regulation, RNA methylation, and symbiont interactions. Regarding cellular components, these genes were enriched in sperm plasma membranes, host cells, and host cellular components. Finally, these genes were found to be enriched in various molecular functions, including heparin binding, phosphoprotein phosphatase activity, and phospholipase activator activity (Fig. 4B, Supplementary Material Table S7).  The KEGG analysis results showed that the main enriched pathways were "Viral protein interaction with cytokine and cytokine receptor", "Chemokine signaling pathway", "Cytokine Cytokine receptor interaction", etc. (Fig. 4C, Supplementary Material Table S8).

### Development and verification of a diagnostic nomogram for IS

**Figure 5:**
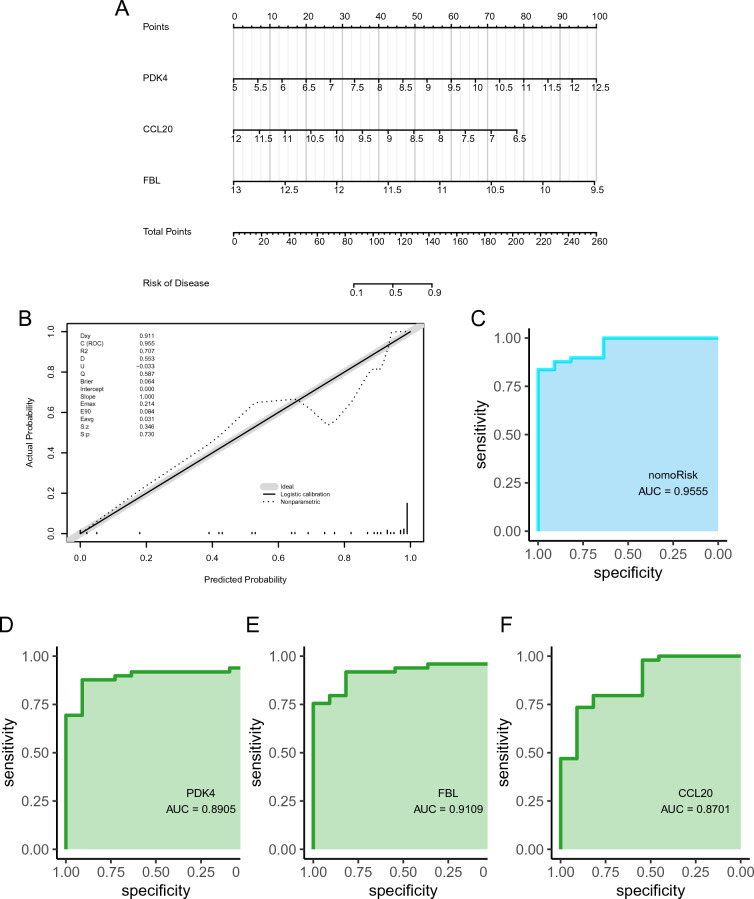
Construction and validation of an IS diagnostic nomogram model. (**A**) IS diagnostic nomogram. (**B**) Calibration curves, and (**C**) ROC curves validate the diagnostic model. (**D**–**F**) ROC curves for (**D**) *PDK4*, (**E**) *FBL*, and (**F**) *CCL20*.

A diagnostic nomogram of IS was developed using the identified feature genes (*PDK4*, *CCL20*, and *FBL*; Fig. 5A), and assessed its predictive power using calibration curves. The calibration curves showed minimal differences between true and predicted IS risk, indicating that the diagnostic nomogram of IS was very accurate (Fig. 5B). Additionally, the three hub genes exhibited good diagnostic value (AUC > 0.8; Fig. 5D–F), with the diagnostic nomogram displaying excellent diagnostic performance (AUC > 0.95).

**Figure 6:**
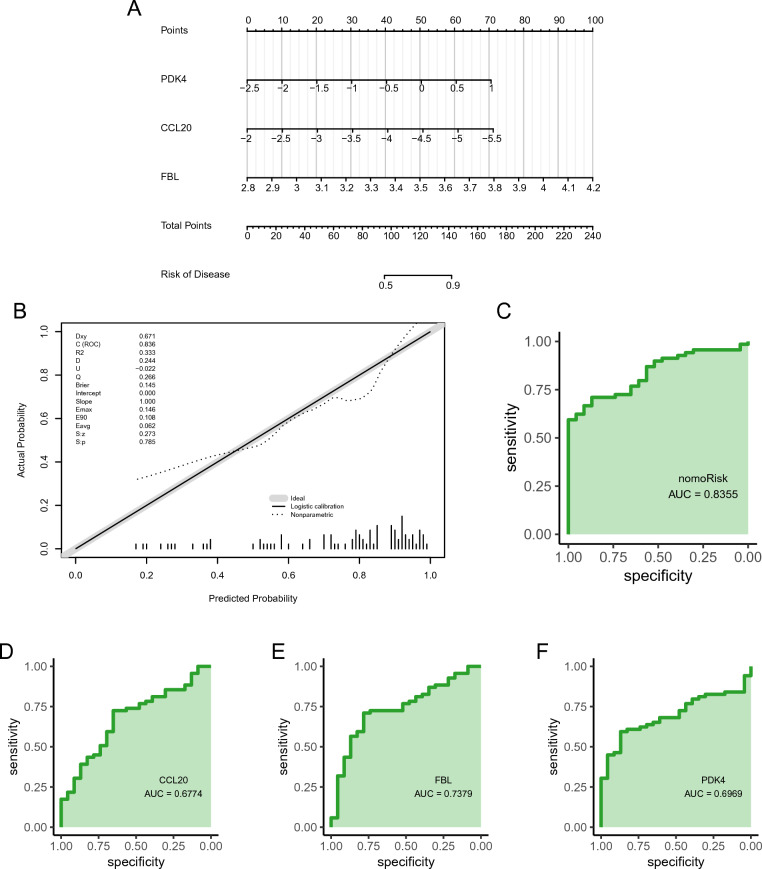
The external validation of an IS diagnostic nomogram model. (**A**) IS diagnostic nomogram. (**B**) Calibration curves, and (**C**) ROC curves validate the diagnostic model. (**D**–**F**) ROC curves for (**D**) *PDK4*, (**E**) *FBL*, and (**F**) *CCL20*.

The external validation dataset (GSE58294) was used to evaluate the diagnostic ability of the feature genes for IS in elderly females (Fig. 6A). Both calibration and validation curves showed that the constructed prognostic model could diagnose IS well in elderly females (Fig. 6B, C). In addition, the ROC of single genes revealed AUCs > 0.6 for *PDK4, CCL20*, and *FBL*, validating the diagnostic efficacy of the feature genes (Fig. 6D–F).

### Immune infiltration

**Figure 7:**
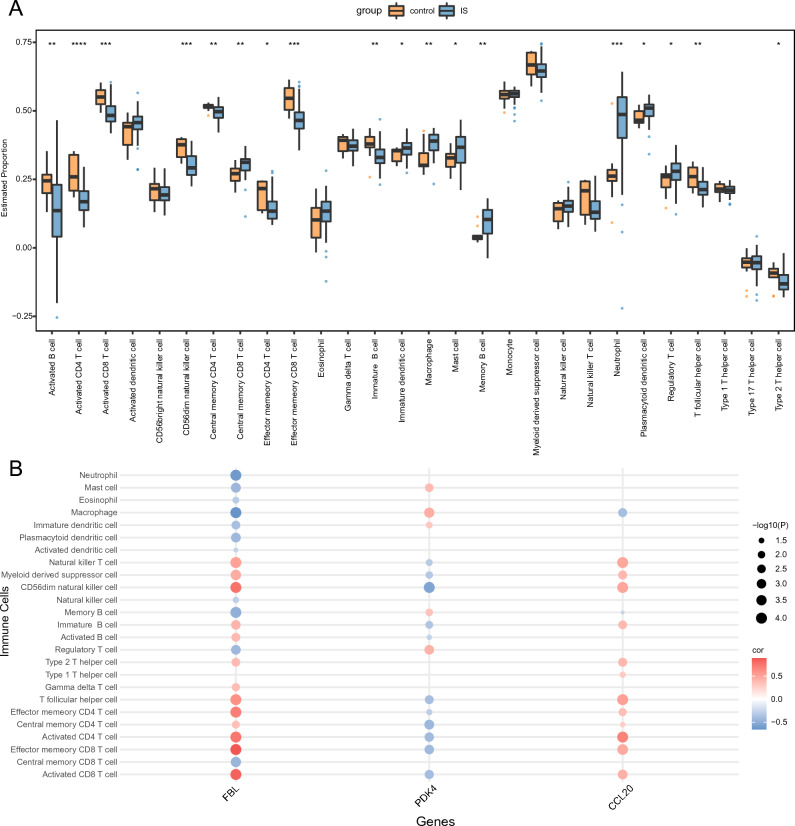
Immune cell infiltration analysis. (**A**) Box plot of immune cell infiltration in IS and control samples. (**B**) Correlation heatmap of immune cell infiltration and hub gene expression.

The correlation between infiltrating immune cells in IS and control samples were evaluated. Levels of infiltrating cells, such as central memory CD8^+^ T cells, immature dendritic cells (DCs), macrophages, mast cells, memory B cells, neutrophils, plasmacytoid DCs, and regulatory T cells, were significantly elevated in the IS group compared to the control group. In contrast, the infiltration levels of activated B cells, effector memory CD8^+^ T cells, activated CD8^+^ T cells, CD56^dim^, activated CD4^+^ T cells, NK cells, central memory CD4 + T cells, effector memory CD4^+^ T cells, immature B cells, T follicular helper cells, and type 2 T helper cells were notably reduced in the IS group relative to the control group (Fig. 7A).

Additionally, the relationships between each hub gene and their respective immune cell type were evaluated (Fig. 7B). The top nine pairings were determined based on absolute correlation values obtained from scatter plots. Notably, *CCL20* exhibited a significant positive correlation with activated CD4^+^ T cells (R = 0.6709, *p* < 0.001; Supplementary Fig. S2A). *FBL* exhibited significant positive correlations with activated CD4^+^ T cells (R = 0.7327, *p* < 0.001, Supplementary Fig. S2B), activated CD8^+^ T cells (R = 0.8341, *p* < 0.001, Supplementary Figure S2C), CD56^dim^ NK cells (R = 0.7562, *p* < 0.001, Supplementary Fig. s2D), effector memory CD4^+^ T cells (R = 0.6994, *p* < 0.001, Supplementary Fig. S2E), effector memory CD8^+^ T cells (R = 0.881, *p* < 0.001, Supplementary Fig. S2F), and T follicular helper cells (R = 0.6083, *p* < 0.001, Supplementary Fig. S2I). Conversely, *FBL* exhibited significant negative correlations with macrophages (R =  − 0.6503, *p* < 0.001, Supplementary Fig. S2G) and neutrophils (R =  − 0.6163, *p* < 0.001, Supplementary Fig. S2H).

### Pan-cancer analysis of hub genes

**Figure 8:**
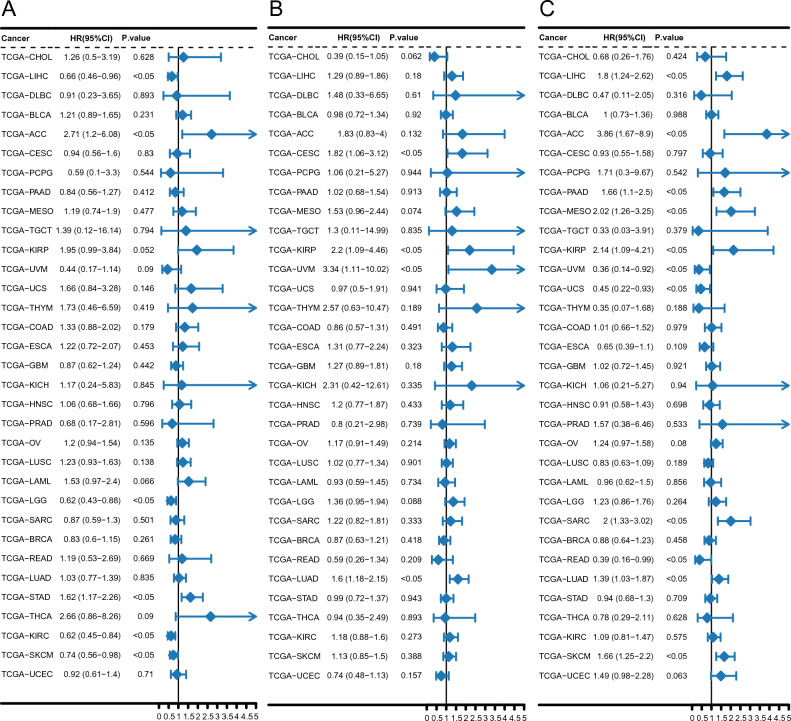
Relationship between overall survival in pan-cancer and hub gene expression. Univariate Cox regression forest plots of (**A**) *PDK4*, (**B**) *CCL20*, and (**C**) *FBL* pan-cancer.

Analysis of the relationship between hub gene expression and the patient prognosis pan-cancer revealed significant findings. Notably, Cox regression analysis, conducted on 33 tumor types, revealed a significant association between PDK4 expression and overall survival in six specific cancers. Specifically, *PDK4* was found to act as a protective factor in liver hepatocellular carcinoma (LIHC), kidney renal clear cell carcinoma (KIRC), low-grade glioma, and skin cutaneous melanoma, while acting as a risk factor in adrenocortical carcinoma and stomach adenocarcinoma (STAD) (Fig. 8A). *CCL20* expression was a risk factor and significantly negatively correlated with overall survival in cervical squamous cell carcinoma, kidney renal papillary cell carcinoma (KIRP), uveal melanoma, and lung adenocarcinoma (LUAD) (Fig. 8B). Finally, in 11 cancer types, *FBL* expression was significantly associated with overall survival, acting as a protective factor in uveal melanoma, uterine carcinosarcoma, and rectum adenocarcinoma (READ), and as a risk factor in LIHC, adrenocortical carcinoma, pancreatic adenocarcinoma, mesothelioma, KIRP, sarcoma, LUAD, and skin cutaneous melanoma (Fig. 8C).

**Figure 9:**
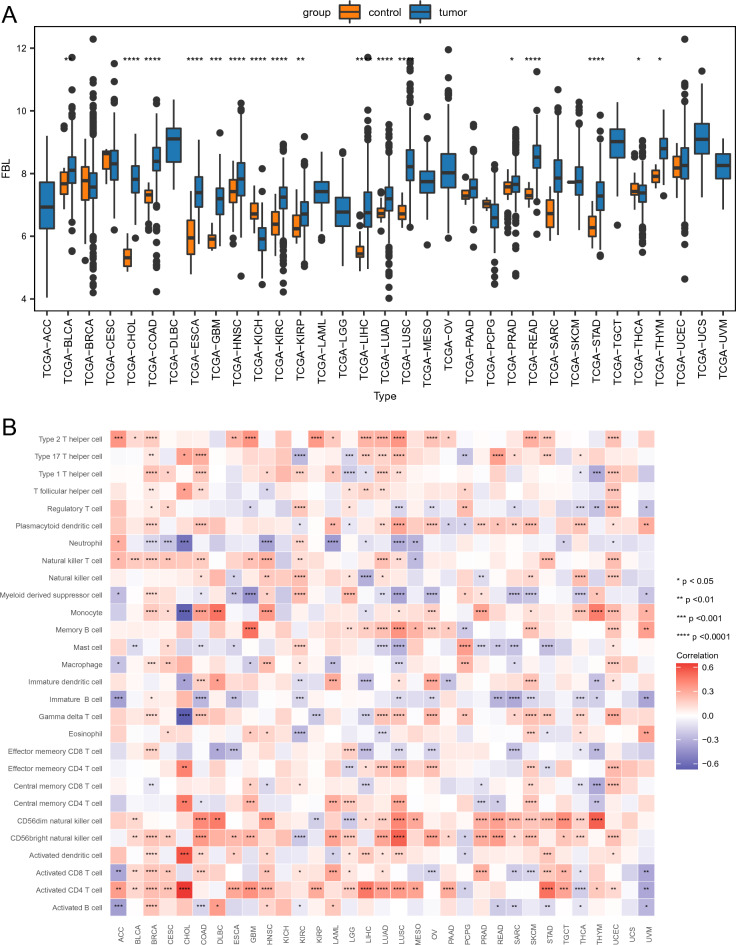
*FBL* expression patterns pan-cancer (**A**) Boxplot of *FBL* expression pan-cancer. (**B**) Heatmap of the correlation between *FBL* and immune infiltration pan-cancer.

Box plots and heat maps were utilized to visualize the relationships between hub gene expression and immune infiltrates pan-cancer. Overall, significantly lower expression levels were observed in bladder urothelial carcinoma, cholangiocarcinoma (CHOL), colon adenocarcinoma (COAD), esophageal carcinoma (ESCA), breast invasive carcinoma, cervical squamous cell carcinoma, head and neck squamous cell carcinoma (HNSC), kidney chromophobe (KICH), pheochromocytoma and paraganglioma, KIRP, prostate adenocarcinoma, LIHC, lung squamous cell carcinoma (LUSC), READ, LUAD, STAD, and uterine corpus endometrial carcinoma (Supplementary Fig. S3A). Notably, *PDK4* expression exhibited the strongest positive correlation with mast cells in LUSC (Supplementary Fig. S3B). Alternatively, significant differences in *CCL20* expression were detected in 14 cancers, with significantly higher expression in breast invasive carcinoma, CHOL, glioblastoma multiforme, COAD, ESCA, HNSC, READ, STAD, KIRC, thyroid carcinoma (THCA), LIHC, LUAD, and uterine corpus endometrial carcinoma, and significantly lower expression in KICH (Supplementary Fig. S4A). *CCL20* expression exhibited the strongest positive correlation with regulatory T cells in THCA (Supplementary Fig. S4B). Finally, significant differences in *FBL* expression were observed in 17 cancers, with significantly higher expression in bladder urothelial carcinoma, READ, COAD, ESCA, HNSC, KIRP, glioblastoma multiforme, KIRC, LIHC, prostate adenocarcinoma, LUAD, CHOL, LUSC, STAD, and thymoma, and significantly lower expression in KICH and THCA (Fig. 9A). Notably, *FBL* exhibited a robust positive correlation with activated CD4 T cells in CHOL (Fig. 9B).

## Discussion

IS arises from the transient or permanent occlusion of cerebral blood vessels, resulting in the eventual death of brain tissue and focal neuronal injury. Owing to the limited therapeutic window and contraindications of treatment, optimal treatment is often not achieved in patients with IS. Additionally, IS trigger an immune response, resulting in neuronal loss and tissue repair^27^. Identifying diagnostic biomarkers and analyzing ICD-associated patterns is crucial for improving prognosis in patients with IS. This study employed bioinformatics analysis to identify diagnostic biomarkers for IS in older females and examin the role of ICD in disease pathogenesis, with aiming of informing the development of targeted treatments.

In this study, 215 IS-associated DEGs related to ICD were identified by taking the intersection between IS-associated DEGs and genes related to ICD. Subsequently, LASSO regression and random forest algorithms were used to screen the hub DEGs related to ICD, resulting in the identification of three hub genes: *CCL20*, *FBL*, and *PDK4*. *CCL20* reportedly exhibits promising potential as a diagnostic biomarker and a therapeutic target in experimental models of cranial brain injury^28^. Furthermore, it has been identified as a central gene in intracerebral hemorrhage^29^ and a key inflammatory response biomarker^30^. Meanwhile, *PDK4* has been proposed as a potential biomarker in patients with renal ischemia^31^ and is considered an important gene related to cell death in females with IS^32^. Moreover, the diagnostic value of a model that incorporates eight inflammation-related genes, including *PDK4*, for the diagnosis of IS was previously reported^33^.

*FBL* encodes a key small nuclear ribonucleoprotein component involved in transcription initiation, carcinogenesis, and apoptosis^34^. Given that the malfunction of ribosomal proteins is associated with disease occurrence and development, these proteins are considered promising therapeutic targets^35^. In particular, a significant positive correlation has been reported between the ribosome pathway activity and the IS incidence^36^. This suggests that ribosomes have a significant function in the development and progression of nervous system disorders. Furthermore, *FBL*-mediated 2ʹ-O-methylation (Nm) of RNA helps cells evade innate immune recognition, maintaining immune homeostasis^37^. This highlights the intricate association between *FBL* and the immune system during disease development, suggesting that *FBL* may represent a promising new target for the diagnosis and treatment of IS.

Overall, these findings indicate that *CCL20, FBL*, and *PDK4* hold promise as potential targets for the diagnosis and treatment of IS. However, the specific effects of these target genes require further experimental validation. Thus, a nomogram model for IS diagnosis was developed, leveraging three hub genes' expression patterns as novel biomarkers. The corresponding ROC curve analysis highlighted the excellent diagnostic performance of the nomogram for predicting IS (AUC = 0.9555). Furthermore, relatively high AUC values were obtained for *PDK4* (AUC = 0.8905), *CCL20* (AUC = 0.9109), and *FBL* (AUC = 0.8701), highlighting their potential as good diagnostic markers for IS.

Using ssGSEA and three hub genes, enriched gene sets in IS samples were identified, with CCL20-like genes significantly enriched in TCR signaling and immunodeficiency pathways, indicating immune dysregulation in IS pathogenesis. GO and KEGG enrichment analysis further revealed significant enrichment of hub genes in pathways involving interactions between virus proteins and cytokines, as well as between cytokines and cytokine receptors. These findings were consistent with the GSEA enrichment results for *PDK4* and *CCL20*.

T and NK cells are associated with neuronal cytotoxicity and inflammation^38^. TCRs play a key role in T cell function and immunological synapse formation, facilitating antigen recognition and immune response initiation. TCR activation can promote various signaling cascades, ultimately influencing cell fate by regulating cytokine production, cell survival, proliferation, and differentiation. NK cells are innate lymphocytes that are essential for host defense against infections. Specifically, NK cells mediate cytotoxicity, an immune defense mechanism that facilitates the elimination of abnormal cells, such as virus-infected cells. However, NK cells exacerbate IS infarction by promoting local inflammation and neuronal hyperactivity^39^. Meanwhile, the interaction between the nervous system and immune system under cerebral ischemia inhibits the NK cells response in the central and peripheral nervous system, mediated by distinct neuroendocrine pathways. Conversely, intracellular pathways in NK cells within the brain and spleen are regulated through a different mechanism^40^. In summary, ICD-associated genes primarily regulate T and NK cell functions, inducing IS onset and progression, revealing novel therapeutic targets.

The interaction between the nervous and immune systems has garnered increasing attention due to its key role in neurodegenerative diseases. The systemic immune alterations and heightened susceptibility to infections following IS contribute to the exacerbation of ischemic damage and long-term disability, leading to poor patients outcomes^34^. Notably, nearly all immune cell populations, including lymphocytes, monocytes, and granulocytes, undergo alterations following stroke^34^. Therefore, immune infiltration was analyzed in the current study within peripheral blood samples from patients with IS. Notably, patients with IS exhibited distinct immune microenvironments in comparison to the control group. Enhanced infiltration of immature DCs, macrophages, mast cells, neutrophils, and plasmacytoid DCs was observed in IS patients versus controls, highlighting their role in IS pathogenesis. Conversely, the infiltration of other immune cells, including activated B cells, activated CD4^+^ T cells, activated CD8^+^ T cells, effector memory CD4^+^ T cells, effector memory CD8^+^ T cells, immature B cells, T follicular helper cells, and type 2T helper cells, were significantly lower in the IS patient group, which aligns with previously reported findings^34,41,42^. Indeed, peripheral blood lymphocyte scores are decreased in patients 24 h post-IS^34^, while the proportion of CD8^+^ T cells is reportedly reduced in female patients with IS^41^. However, elevated infiltration of central memory CD8^+^ T cells and regulatory T cells in patients with IS compared to controls indicates complex immune interactions in IS pathogenesis. T-cells play a key role in initiating thrombotic inflammation by facilitating leukocyte adherence to the endothelium of the cerebrovascular system^43^. Post-stroke T-cell infiltration into the brain aggravates tissue damage via inflammation, emphasizing the need for immune-modulating therapies in stroke treatment^44^. CD8^+^ T cells, i.e., cytotoxic T lymphocytes, kill target cells via secretion of cytolytic mediators, such as perforin and granzyme, which induce apoptosis, and cytokines, such as interferon-gamma, and tumor necrosis factor-alpha that increase antigen presentation and further increase target cell killing. Meanwhile, central memory CD8^+^ T cells are resident lymphocytes that respond to secondary infections through rapid proliferation.

In the present study, a heightened degree of macrophage, mast cell, and neutrophil infiltration was observed in IS. Specifically, the proportion of M1 macrophages and the macrophage score were elevated in the peripheral blood of patients with IS patient compared with controls, demonstrating their key role in IS pathogenesis^45^. Similarly, female patients with IS were reported to exhibit increased proportions of M0 macrophages, M macrophages, and neutrophils^41^. Infiltrative inflammatory cells, including neutrophils, macrophages, and lymphocytes, exacerbate the progression of ischemic injury.

The current study also revealed a considerably increased degree of immature and plasmacytoid DC infiltration in patients with IS compared with the control group. DCs bridge the innate and adaptive immune responses. Notably, the abundance of intracerebral DCs reportedly increases following permanent middle cerebral artery occlusion; moreover, DC abundance correlates with the extent of brain injury, indicating that DCs play a key role in cerebral ischemia^46^.

In the present study, pan-cancer analysis identified *PDK4*, *CCL20*, and *FBL* as central genes (hub genes) with significant differences in expression in 17, 14, and 17 cancer types, respectively. Additionally, a previous study suggested that stroke occurrence may serve as an early indicator of tumorigenesis^47^. Notably, the gene expression profiles associated with ischemic injury not only correlate with overall cell death and critical cancer pathways but also exhibited strong associations with various tumor types. Furthermore, the expression of these gene sets following a stroke has been linked to an increased risk of death^47^. Therefore, *CCL20, FBL*, and *PDK4* emerged as common biomarkers for IS and pan-cancer, crucial for predicting prognosis in patients with both conditions. Overall, these findings will help facilitate the effective diagnosis and treatment of these diseases, offering promising strategies for improving patient survival rates and quality of life.

Although this study offers valuable insights, it is important to acknowledge several limitations. First, the data primarily relied on internet databases, potentially limiting the representativeness and comprehensiveness of the findings. To enhance the reliability and applicability of future studies, it is essential to increase sample sizes and incorporate data from various geographical regions and populations. Secondly, the key IS biomarkers identified in our study have yet to be validated in a laboratory setting. Therefore, further confirmation of the efficacy and reliability of these biomarkers via preclinical and clinical trials is necessary. The overall aim of these studies will be to develop more accurate diagnostic methods, effective preventive strategies, and targeted treatment plans for older female patients with IS, improving their prognosis and quality of life.

## Conclusions

This study highlights the key roles of ICD-related genes in various biological processes. Through comprehensive bioinformatics analysis, DEGs associated with ICD in older female patients with IS were identified and evaluated, hub genes were detected, and diagnostic nomogram was construction Ultimately, *CCL20, FBL*, and *PDK4* were dentified as key diagnostic biomarkers in older female patients with IS. This study provides a novel and comprehensive perspective on the involvement of ICD in the pathogenesis of IS in older females. Furthermore, it offers new avenues for future research on immunoregulation in IS and contributes to the advancement of therapeutic strategies for the treatment of cerebrovascular diseases.

### Supplementary Information

Supplementary Figure S1.

Supplementary Figure S2.

Supplementary Figure S3.

Supplementary Figure S4.

Supplementary Table S1.

Supplementary Table S2.

Supplementary Table S3.

Supplementary Table S4.

Supplementary Table S5.

Supplementary Table S6.

Supplementary Table S7.

Supplementary Table S8.

## Data Availability

All data from the GSE37587, GSE22255, GSE16561, and GSE58294 datasets are free and open access, and they are obtained from GEO (https://www.ncbi.nlm.nih.gov/geo/).
