# Supplementary figures and images for: Potential diagnostic biomarkers for immunogenic cell death in elderly female patients with ischemic stroke: identification and analysis
Source: Sci Rep. 2024 Jun 24;14:14553. doi: 10.1038/s41598-024-65390-w (PMC11196739; doi:10.1038/s41598-024-65390-w)

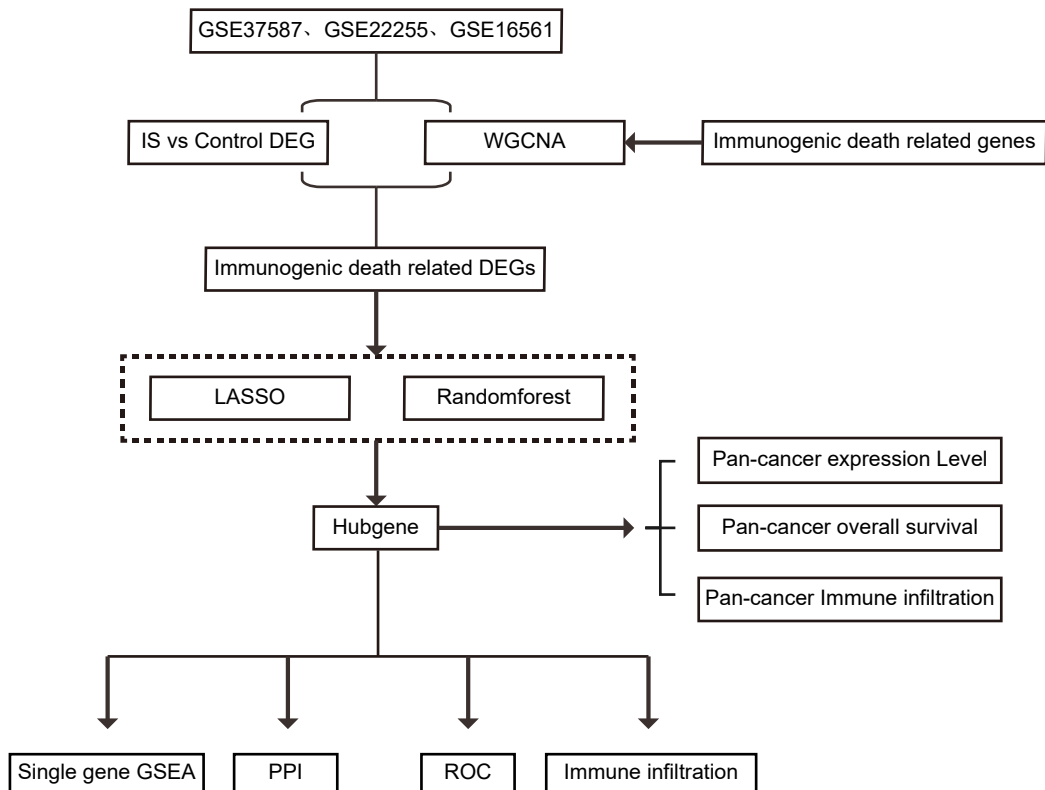

Supplement: Supplementary file 1 — Supplementary Figure S1. [file 41598_2024_65390_MOESM1_ESM.pdf]

A

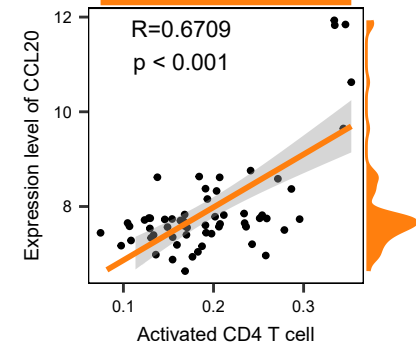

B

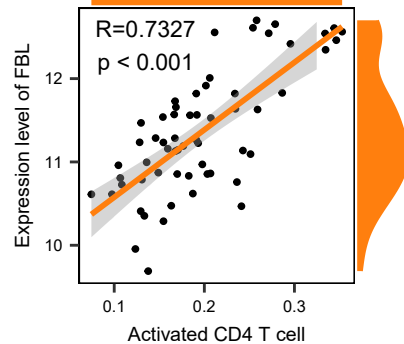

C

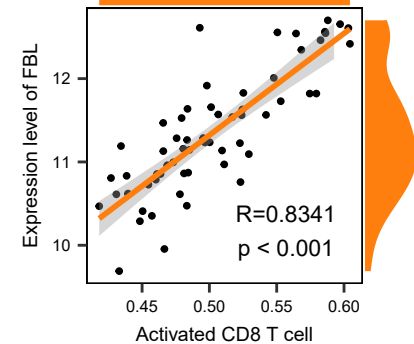

D

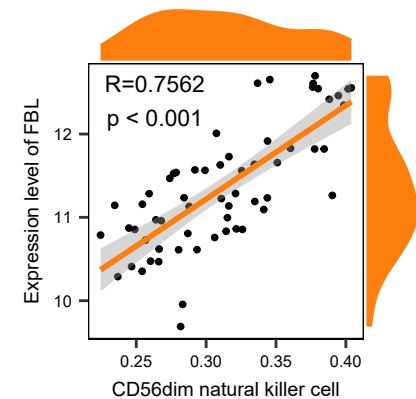

E

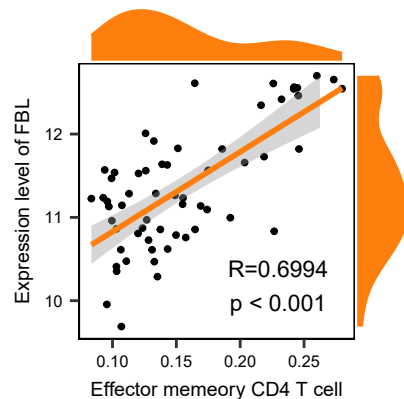

F

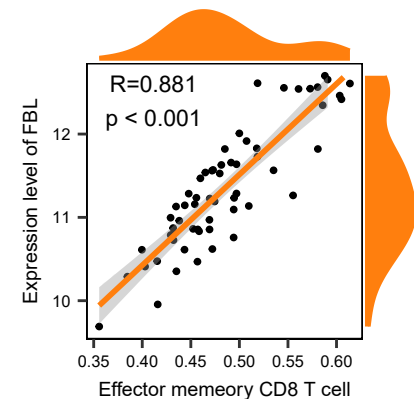

G

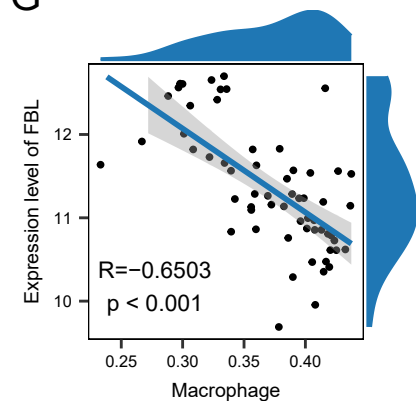

H

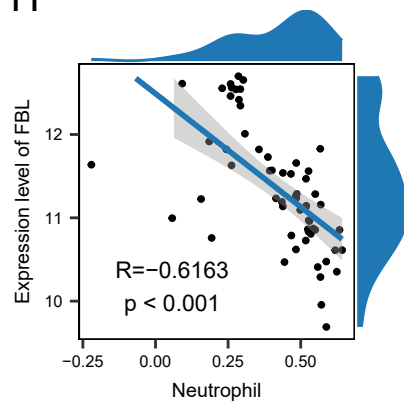

I

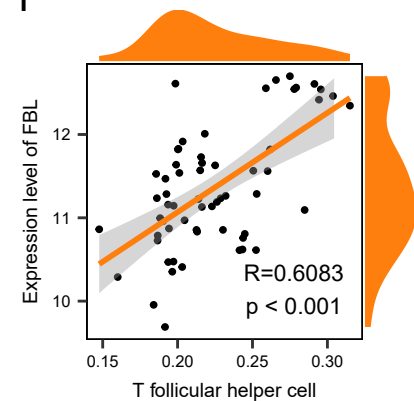

Supplement: Supplementary file 2 — Supplementary Figure S2. [file 41598_2024_65390_MOESM2_ESM.pdf]

A

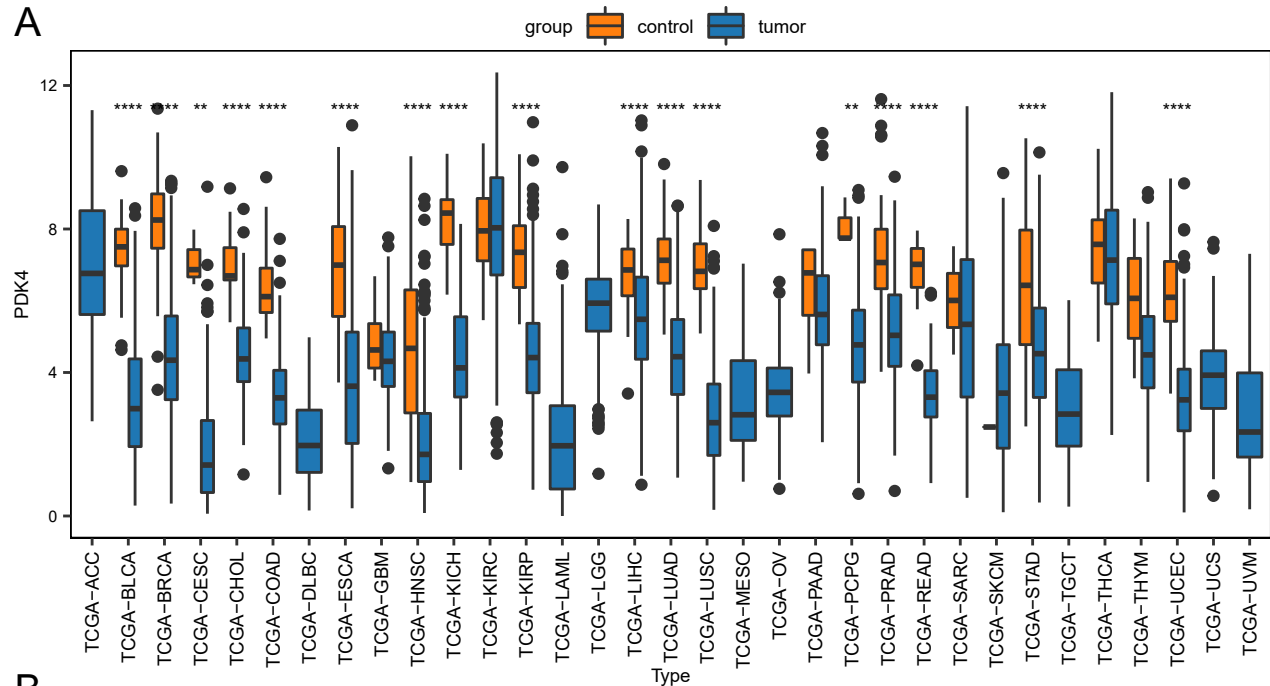

B

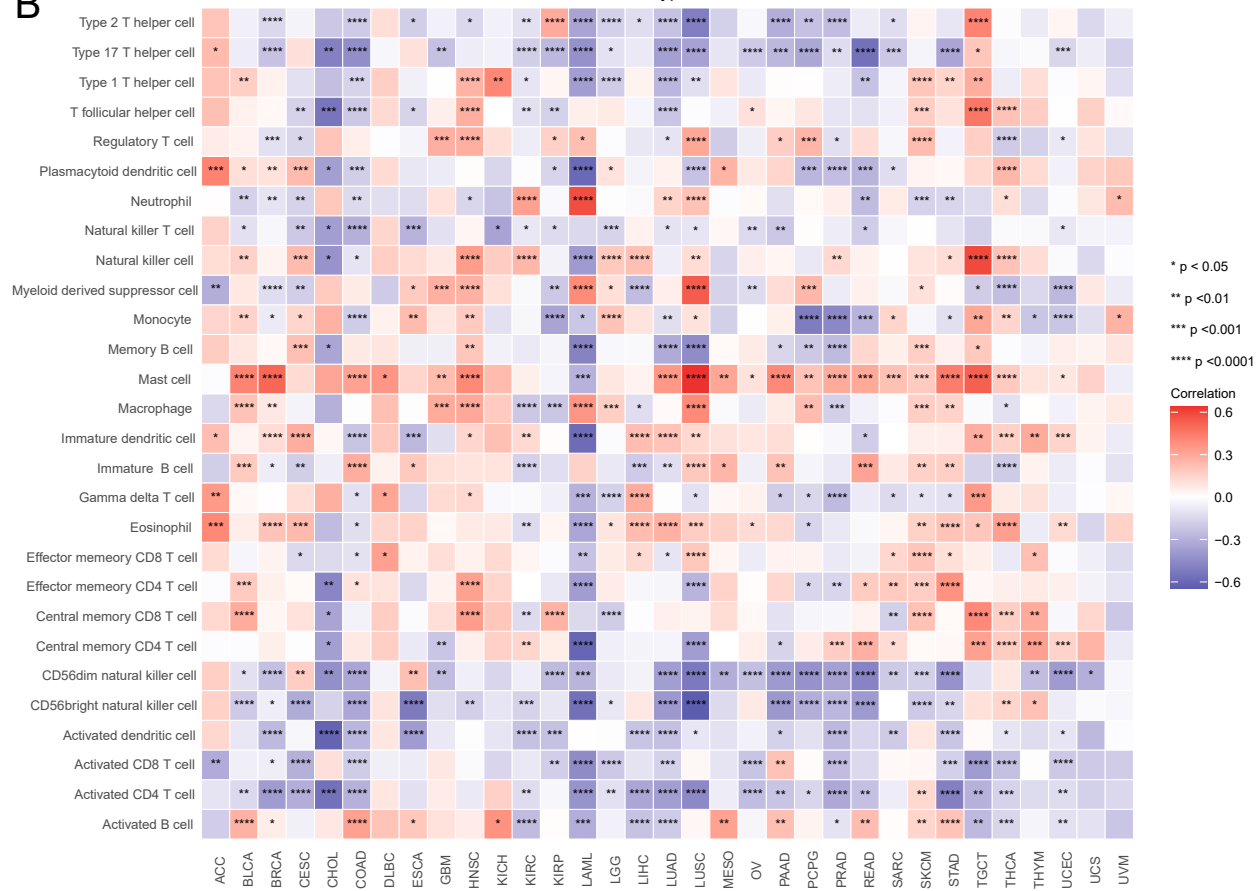

Supplement: Supplementary file 3 — Supplementary Figure S3. [file 41598_2024_65390_MOESM3_ESM.pdf]

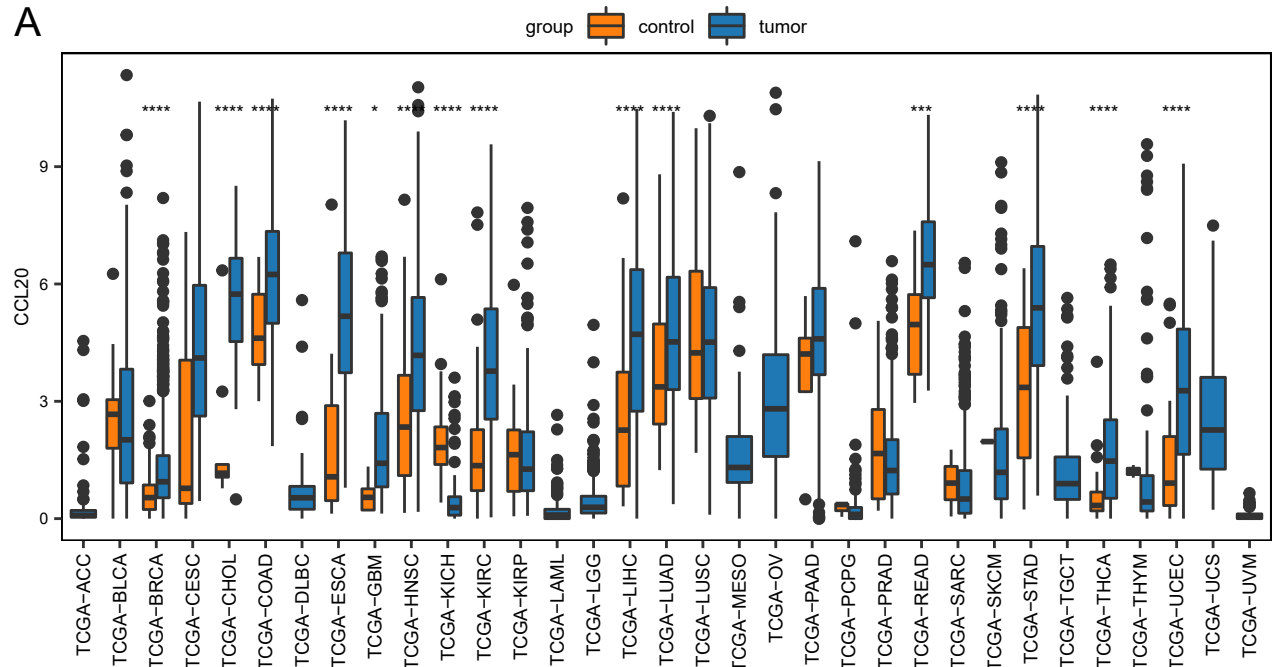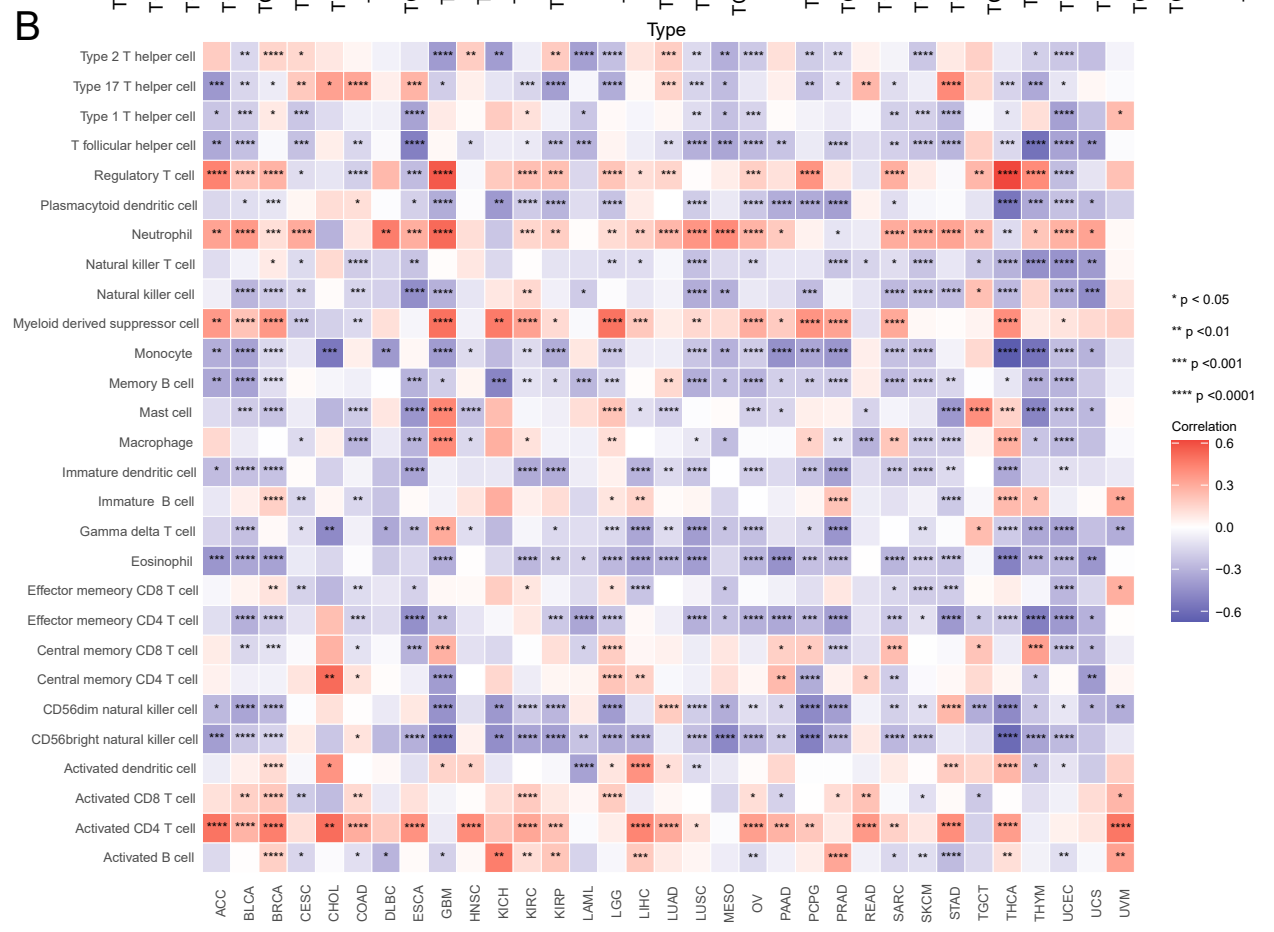

Supplement: Supplementary file 4 — Supplementary Figure S4. [file 41598_2024_65390_MOESM4_ESM.pdf]
